# Moving prevention of functional impairment upstream: is middle age an ideal time for intervention?

**DOI:** 10.1186/s40695-020-00054-z

**Published:** 2020-07-17

**Authors:** Rebecca T. Brown, Kenneth E. Covinsky

**Affiliations:** 1 Division of Geriatric Medicine, Perelman School of Medicine at the University of Pennsylvania, 3615 Chestnut Street, Philadelphia, PA 19104 USA; 2 Geriatrics and Extended Care, Corporal Michael J. Crescenz Veterans Affairs Medical Center, Philadelphia, PA USA; 3 Center for Health Equity and Research Promotion, Corporal Michael J. Crescenz VA Medical Center, Philadelphia, PA USA; 4 Division of Geriatrics, University of California, San Francisco, CA USA; 5 San Francisco Veterans Affairs Medical Center, San Francisco, CA USA

**Keywords:** Middle age, Functional limitation, Physical functioning

## Abstract

To live independently, individuals must be able to perform basic activities of daily living (ADLs), including bathing, dressing, and transferring out of a bed or chair. When older adults develop difficulty or the need for help performing ADLs, they experience decreased quality of life and an increased risk of acute care utilization, nursing home admission, and death. For these reasons, slowing or preventing the progression to functional problems is a key focus of the care of older adults. While preventive efforts currently focus mainly on older people, difficulty performing basic ADLs (“functional impairment”) affects nearly 15% of middle-aged adults, and this prevalence is increasing. People who develop functional impairment in middle age are at increased risk for adverse outcomes similar to those experienced by older adults. Developing ADL impairment in middle age also impacts work force participation and health expenditures, not just in middle age but also older age. Middle-aged adults have a high capacity for recovery from functional impairment, and many risk factors for developing functional impairment in middle and older age have their roots in mid-life. Taken together, these findings suggest that middle age may be an ideal period to intervene to prevent or delay functional impairment. To address the rising prevalence of functional impairment in middle age, we will need to work on several fronts. These include developing improved prognostic tools to identify middle-aged people at highest risk for functional impairment and developing interventions to prevent or delay impairment among middle-aged people. More broadly, we need to recognize functional impairment in middle age as a problem that is as prevalent and central to health outcomes as many chronic medical conditions.

## Background

To live independently, individuals must be able to perform basic activities of daily living (ADLs), including bathing, dressing, toileting, transferring out of a bed or chair, and feeding oneself. As people age, they may develop difficulty performing these activities, or “functional impairment.” Over time or with the onset of illness, individuals may progress from having difficulty with daily activities to needing help from another person to perform these activities, often called “disability.” When older adults develop functional impairment or disability, they experience decreased quality of life and an increased risk of acute care utilization, nursing home admission, and death [1–3]. For this reason, a large body of research has focused on identifying older adults at highest risk for developing ADL problems and developing and testing interventions targeted to those at highest risk. This research has focused almost exclusively on people ages 70 or older, and has shown that targeted interventions can prevent ADL problems among high-risk adults in this age group [4–6].

Yet a growing body of research shows that functional impairment is also common in middle-aged people (i.e., ages 45–64), and that the prevalence of functional impairment is increasing in this age group [7–12]. From 2000 to 2008, the prevalence of difficulty performing one or more ADLs increased from 15 to 16% among people aged 55–64 [9], an increase of approximately 420,000 people, and this upward trend continued through 2016 [12]. While needing help with ADLs remains relatively rare in middle age [7, 9, 11, 12], developing difficulty with these activities represents a key step on the pathway towards needing help and strongly predicts adverse outcomes [13, 14]. Recent research shows that developing ADL difficulty in middle age is associated with outcomes similar to those seen in late life, including hospitalization, nursing home admission, and death [13]. Developing ADL impairment in middle age also impacts work force participation and health care utilization and expenditures, not just in middle age but also older age [7, 15]. Yet while research among older adults has advanced from identifying those at risk to implementing interventions to prevent functional impairment, we lack similar progress to address functional impairment among middle-aged people.

In this commentary, we will discuss why middle age may be an ideal time to intervene to prevent or delay the development of functional impairment in both middle age and older age. We will also consider what types of interventions may be most effective in this age group.

## Main text

### Epidemiology and outcomes of functional impairment in middle age

#### Functional impairment is becoming more common in middle age

A growing body of research shows that over the past two decades, health has worsened among middle-aged people in the United States. This includes increases not only in the prevalence of functional impairment but also in the prevalence of chronic conditions such as diabetes, obesity, and arthritis [16] which are important causes of functional impairment in the United States [7, 17]. The reasons for worsening health and function in middle age are complex and an area of active study. Changing health behaviors play an important role in the increased prevalence of chronic conditions, including increases in caloric intake, changes in dietary composition, and decreasing levels of physical activity [18]. However, the central role of socioeconomic status (SES) is increasingly recognized. Socioeconomic disparities in mortality risk and prevalence of chronic conditions have worsened substantially over the past several decades in the U.S., a trend occurring in parallel with economic stagnation for lower-income households [19, 20]. Based on these trends, researchers hypothesize that lower SES is leading to worsening health in middle age, possibly through the negative impact of lower SES on factors such as environment, access to health care, and stress [21–23]. Research using data from the 2002–2016 National Health Interview Survey supports this hypothesis, showing how unfavorable trends in income and psychological distress are associated with worsening function over time, while improving educational attainment and decreasing rates of smoking are protective factors [12]. Given these trends towards worsening health and function among middle-aged people, there are concerns that without action, this age cohort will enter older age in worse health than the current cohort of older adults [21].

#### Functional impairment in middle age is heterogeneous

Many studies of middle-aged people with ADL impairment are cross-sectional and capture a heterogeneous group of individuals [7, 9, 10]. This group includes people with long-standing impairments that are congenital or developed in young adulthood, as well as people with impairments that developed for the first time in middle age. Research shows that both of these groups experience poorer health outcomes than people without such impairments. Individuals with longstanding disabilities are at disproportionate risk for poor health outcomes [24, 25], while people who develop new ADL impairment in middle age have a 1.5- to 2.5-fold increased risk of hospitalization, nursing home admission, and death [13, 14]. These groups represent distinct populations, with different pathways to functional impairment and different needs, for whom different interventions may be appropriate. In this commentary, we will focus specifically on people who develop functional impairment for the first time in middle age.

#### Functional impairment that develops in middle age has similarities to impairment that develops in older age, but also key differences

Some risk factors for ADL impairment in middle and older age are similar, including having lower income, chronic medical conditions, depression, sensory impairment, low physical activity, reduced physical performance, and living in an unsafe neighborhood [26–30]. However, some established risk factors among older adults do not appear to be risk factors in middle-aged people, including female sex, cognitive impairment, and low body mass index (Table 1) [13, 14, 31–33]. Several factors may explain these differences. Older women are thought to be at increased risk for ADL impairment compared to men because they have a higher prevalence of disabling conditions, including osteoporosis and osteoarthritis [34–38]. These conditions do not become highly prevalent until age 65, when gender differences in risk of ADL impairment emerge [37]. However, middle-aged women have a higher prevalence of risk factors for ADL impairment than men. These risk factors include greater declines in strength [39–41] and lower scores on physical performance tasks including grip strength and chair rise [42, 43]. Greater loss of strength in women compared to men may result from the menopausal transition [41, 44]. Cognitive impairment strongly predicts ADL impairment among older adults but is relatively rare in middle age, making it challenging to examine its association with functional outcomes; the same is true for low body mass index [14].

**Table 1:** Risk factors for ADL impairment by age group

Middle age and older age	Older age only
Low income	Female sex
Chronic medical conditions (e.g., stroke, diabetes, arthritis, lung disease, obesity)	Cognitive impairment
Depression	Low body mass index
Sensory impairment	
Reduced physical performance	
Low physical activity	
Living in an unsafe neighborhood	

Several studies also suggest that the traditional “hierarchy of disability” observed among older adults may differ in middle-aged people. When older adults develop difficulty performing ADL tasks, these difficulties develop in a predictable order, often called a hierarchy of disability [45]. Tasks requiring strength, balance, and coordination, such as bathing and dressing, are affected first, while tasks that require manual dexterity, such as eating, are affected later [45–49]. However, the order of onset of ADL impairments differs in middle-aged adults, with transferring and walking across a room being the most common initial impairments in this age group [14, 50, 51]. Additionally, in middle-aged adults, impairments in cognitively complex instrumental activities of daily living (IADLs) – such as managing medications and shopping – appear to be less common than ADL impairments [46, 52]. In older adults, in contrast, IADL impairments typically precede ADL impairments [13, 14]. The reasons for this difference by age group are not yet clear. Among older adults, cognitive impairment impacts the ability to perform cognitively-complex IADL tasks [45, 53]. In middle-aged people, the lower incidence of IADL impairment may reflect the lower prevalence of cognitive impairment in this age group. Taken together, these differences suggest that the pathogenesis of ADL impairment differs by age, and point to the need to develop interventions that are tailored specifically to middle-aged people.

#### Functional impairment and disability in middle age impacts work force participation and public expenditures

Functional impairment and disability that develops before age 65, often called pre-retirement age disability, has major impacts on work force participation and public expenditures. In 2018, 6.8 million disabled workers ages 45–64 in the U.S. received disability benefits [54]. Individuals who develop disability experience substantial economic effects; one study showed that 10 years after onset of disability, people with chronic and severe disability had average declines of 79% in earnings and 35% in after-tax income; the average disabled worker experienced economic impacts about half that of individuals with severe and chronic disability [15]. Federal disability benefits for disabled workers include Social Security Disability Insurance and Supplemental Security Income programs and health insurance via Medicare and Medicaid. Public expenditures for these benefits exceed $150 billion annually [55]. The number of disabled workers has recently fallen, a trend which may be related to the economic recovery and the shift towards jobs that require less physical labor [56]. However, worsening income inequality and health disparities may stall or reverse these positive trends [56].

### Middle age may be an ideal time to intervene to prevent or delay the onset of functional impairment and mitigate associated burdens and costs

Several lines of research suggest that middle age is a promising time for intervention to delay or prevent functional impairment. First, middle-aged people who develop ADL impairment appear to have a higher capacity for improvement than do older adults. Studies show that ADL impairment in both middle age and older age is dynamic, with many people experiencing improvement in function after an initial episode of impairment [14, 57, 58]. However, the proportion of people who improve after an initial episode of impairment is higher among middle-aged than older adults. For example, in a prospective nationally representative study of men and women in the U.S. who were ages 50–56 at study enrollment, 22% developed new ADL impairment before age 65. Of individuals with new ADL impairment, 65% had stable or improved function at 10 year follow-up; risk of functional decline was similar in women versus men [14]. By contrast, in a separate prospective population-based study of men and women aged 85 or older in the Netherlands, only 14% of participants who developed new ADL impairment had stable or improved function at 5 years [58, 59]. Men were less likely to decline over the follow-up period than were women (hazard ratio for decline, 0.75 [95% CI, 0.58–0.96]). Other population-based studies similarly show that the probability of recovery from disability decreases with increasing age [60, 61]. These findings suggest that with appropriate interventions, middle-aged adults at high risk for impairment may be more likely to maintain independence than older adults. The greater resilience of middle-aged versus older adults may reflect in part changes in muscle that occur with aging and which may be accelerated in women during the menopausal transition, including loss of mass, strength, and function, termed sarcopenia [62]. Some theorize that because middle-aged adults have less sarcopenia than older adults, exercise interventions that prevent ADL impairment in older adults may be even more effective for middle-aged people [60, 63].

Addressing ADL impairment earlier in the life course is also a promising strategy for proactively addressing the burdens and costs of ADL impairment, including impacts on work force participation and public expenditures. A growing body of research shows that functional impairment in both middle-aged and older adults have their roots in conditions that start in middle age, including arthritis, obesity, and depression [64–66]. Intervening in middle age thus has the potential not only to reduce the incidence of ADL impairment and associated adverse outcomes among middle-aged people, but also older people. This approach aligns with a growing consensus that achieving healthy aging requires a life-course approach to promoting health and function that starts decades before we enter traditional older age [67, 68]. Taking a proactive approach to preventing functional impairment is even more pressing as the population of middle-aged people with functional impairment grows [12, 21, 22].

### Potential challenges to intervening in middle age

To address functional impairment among the growing population of at-risk middle-aged people, we must consider several potential challenges.

#### Challenge 1: dynamism: functional impairments come and go

One potential challenge is the dynamic nature of functional impairment in middle age. While functional impairment is also dynamic in older adults, it appears to be especially so in middle-aged adults, with a higher proportion of initial impairments improving among middle-aged compared to older people [14, 26, 59]. However, recent research among middle-aged people shows that an initial episode of functional impairment strongly predicts adverse outcomes including hospitalization, nursing home admission, and death, regardless of whether that impairment later regresses [13, 14]. This finding is consistent with research among older adults showing than an initial episode of functional impairment represents a “sentinel event” signaling the risk for future adverse outcomes [3, 61, 69–71]. Given that an initial episode of impairment has similar prognostic importance for middle-aged people, preventing an initial episode of functional impairment is also an important goal in this younger age group [13, 14].

#### Challenge 2: follow-up time

Another potential challenge is the long follow-up time that may be needed to observe the effectiveness of an intervention to prevent or delay functional impairment [60]. For interventions focused on preventing functional impairment in middle age, this is less of a concern as long as accurate prognostic models are available to identify those at highest risk for developing functional impairment in middle age. However, this is a challenge for life course interventions designed to intervene in middle age to prevent or delay functional impairment or other outcomes in older age. Such trials require long follow-up periods and associated challenges of participant retention and costs. Potential strategies for addressing these challenges include initially focusing on upstream outcomes that are strongly correlated with disability, including performance measures such as gait speed [72] and the Short Physical Performance Battery [73], or self-reported physical capacity [74]. However, it is important that these trials also incorporate methods for long-term follow-up of functional outcomes.

In the absence of randomized controlled trials, observational studies provide important evidence about the association of risk factors in middle age with late-life outcomes, while simulation studies model the impact of healthy life style changes in middle age (e.g., weight loss, quitting smoking) on late life health [75]. In the field of dementia prevention, the World Health Organization and others have used such evidence to develop evidence-based guidelines for life course approaches to reducing dementia incidence [76, 77]. While some studies have examined the most efficacious late-life interventions to prevent disability [78], to our knowledge, we currently lack similar life course guidelines to prevent or delay functional impairment and disability.

#### Challenge 3: potential similarities between interventions to prevent functional impairment and existing preventive care interventions

Another potential challenge is the perception that there is no added benefit to developing new interventions to prevent or delay functional impairment for middle-aged people, because existing primary care interventions already address risk factors for functional impairment. Currently, primary care for middle-aged adults focuses on addressing cardiovascular risk factors such as high blood pressure, performing screenings for cancer, diabetes, and other conditions, and targeting high-risk patients for prevention interventions such as statins. Preventive care for adults of all ages focuses on promoting healthy behaviors including physical activity, healthy diet, and smoking cessation. Many of these primary care interventions target risk factors that are also risk factors for functional impairment, including chronic health conditions and health-related behaviors.

However, several lines of research point to the importance of developing tailored interventions that specifically focus on preventing functional impairment in middle age. First, despite our health system’s current focus on modifying cardiovascular risk factors and promoting healthy behaviors, the prevalence of functional impairment in middle age continues to increase. This suggests that our current clinical approaches are not enough to reverse trends towards worsening health in middle age. Second, functional impairment in both middle-aged and older people is multifactorial, meaning that these impairments result from the interaction of risk factors from multiple domains, including sociodemographics, health status, health-related behaviors, and the physical environment [13, 14, 53]. Thus, it is likely that interventions to address functional impairment will need to be multifactorial and coordinated, addressing multiple risk factors in concert. Third, individuals who develop functional impairment in middle age tend to be especially vulnerable, with a high prevalence of risk factors for poor health [13, 14, 50]. To improve outcomes for this high-risk group, a targeted approach may be most efficacious, rather than a diffuse approach that provides similar prevention advice to all patients. Last, function is the outcome that is most important to older adults [79]. Thus, interventions to prevent functional impairment may be especially motivating for patients because they align with their larger goals.

### Future directions and opportunities

A robust body of research among older adults provides a framework to help inform and advance the science of functional impairment prevention among middle-aged people. Among older adults, research has focused on two key areas: first, identifying older adults who are at highest risk for functional impairment, and second, developing and testing interventions to prevent or delay impairment among those at highest risk [5]. This research shows that functional impairment among older adults is multifactorial, with risk factors encompassing demographics, socioeconomic status, health status, health-related behaviors (smoking, low physical activity), geriatric conditions (cognitive impairment, sensory impairment, urinary incontinence), social support, and environment (neighborhood safety, walkability) [53, 80–82]. Researchers have used these findings to develop multifactorial prognostic models that are highly accurate in identifying those at highest risk [73, 83–85]. In turn, these risk factors and indices have informed a growing number of interventions to mitigate, prevent, or delay functional impairment among high-risk older adults [5, 6]. For example, a structured, moderate-intensity physical activity program targeted to high-risk adults ages 70 and older reduced incident mobility disability by 18% [4], and a multi-component home-based intervention including occupational therapy, nursing, and handyman visits decreased disability by 30% among high-risk older adults [86, 87].

This research among older adults has several implications for prevention of functional impairment among middle-aged people. Current health care for middle-aged people focuses mainly on prevention of chronic illnesses, such as heart disease and diabetes. Preventing chronic conditions that are risk factors for functional impairment is a key part of a proactive strategy to prevent functional impairment in middle age. However, risk factors for functional impairment in middle age span a broad range of domains, including not just chronic conditions but also sensory impairments and social and environmental risk factors. Thus, to address the increasing prevalence of functional impairment among middle-aged people, we need to adjust current paradigms to include prevention of functional impairment as an important clinical goal. To do so, we need to move prevention beyond a model that thinks only in terms of specific diseases and towards a broader, more holistic view of health that also considers function.

Accurate prognostic tools that can identify those at highest risk for functional impairment will help achieve this goal while minimizing burden for busy primary care providers. While prognostic tools exist to identify people ages 50 and older at risk for functional impairment [30], risk factors for functional impairment differ in middle versus older age [14]. For this reason, predictive models that focus specifically on middle-aged adults are likely to be able to identify and appropriately weight risk factors that are most important in this age group, leading to a more accurate index for middle-aged people. Additionally, clinical guidelines that build on existing epidemiologic research about risk factors for disability could help guide patients and providers in how to maintain and improve function in middle and older age, while we await more targeted interventions.

Interventions shown to be effective in preventing or reducing functional impairment among older adults provide a template for interventions for middle-aged people. The Lifestyle Interventions and Independence for Elders (LIFE) intervention was a multicenter randomized trial of a long-term structured physical activity program that was targeted to older adults (ages 70–89 years) at high risk for mobility disability [4]. The intervention incorporated twice-weekly center-based exercise sessions including aerobic, resistance, and flexibility training. Over 2.6 years’ follow-up, individuals randomized to the intervention versus a health education program had an 18% lower risk of developing persistent mobility disability. Another recent trial is the Community Aging in Place – Advancing Better Living for Elders (CAPABLE) study, a randomized trial of a 10-session, home-based, multidisciplinary program including visits by an occupational therapist, nurse, and handyman [86, 87]. This trial enrolled a vulnerable population of low income, community-dwelling older adults with baseline disability and focused on addressing not just deficits related to the person’s capability, but also those related to environmental demand and adaptation. The multidisciplinary team conducted assessments with participants to identify their goals and unmet health and environmental needs and developed tailored plans to address those goals and needs. Over 5 months, participants in the intervention group had a 30% reduction in ADL disability scores compared to a control group receiving home visits from a research assistant.

Both of these approaches may inform interventions to prevent or mitigate functional impairment among middle-aged adults (Table 2). For middle-aged people whose main risk factor for functional impairment is declining strength and physical performance, an intervention focused on exercise, including aerobic, resistance, and flexibility training, may be effective. An exercise-focused approach may be particularly appropriate for women at risk for functional impairment, given greater declines in strength and physical performance among women compared to men [43–45]. However, for socioeconomically disadvantaged populations which experience health disparities and have a greater burden of health, social, and environmental risk factors, a multi-component approach such as CAPABLE may be most appropriate. Indeed, CAPABLE is already being adapted for a high-risk population of formerly homeless adults [88], showing the potential to adapt this intervention for vulnerable middle-aged groups. Given evidence that worsening socioeconomic status is associated with the rising prevalence of functional impairment in middle-aged people [21, 22], interventions such as CAPABLE have the potential to play an important role in mitigating these trends.

**Table 2:** Promising intervention components to prevent, delay, or mitigate functional impairment in middle-aged adults

Intervention components	Exemplar intervention for older adults and impact	Target population
Structured exercise programs including aerobic training, resistance training, and flexibility training	Lifestyle Interventions and Independence for Elders (LIFE) Study Randomized Clinical Trial; reduced risk of major mobility disability in at-risk older adults	May benefit middle-aged individuals whose main risk factor for functional impairment is reduced strength and physical performance, including post-menopausal women
Multi-component interventions addressing deficits related to physical capability and to environmental demand and adaptation	Community Aging in Place – Advancing Better Living for Elders (CAPABLE) study; reduced ADL disability scores in at-risk group	May benefit middle-aged individuals with multiple risk factors for functional impairment, including reduced physical performance and social and environmental risks

Adaptations of existing interventions will also need to account for differences between middle-aged and older adults. These include the differing epidemiology of functional impairment in middle-aged people and differences in life stage between middle-aged versus older adults. For example, a larger percentage of middle-aged people work compared to older adults. In 2018, 80% of people ages 45–54 and 65% of people ages 55–64 participated in the U.S. civilian work force [89]. In contrast, 27% of people ages 65–74 and just 8.7% of those aged 75 and older worked in 2018. A substantial percentage of middle-aged adults also care for children or parents [90]. Thus, the frequency or timing of interventions may need to be adjusted, and mobile health approaches and other flexible intervention delivery platforms may need to be considered.

## Conclusions

The prevalence of functional impairment among middle-aged Americans is increasing. Functional impairment in this age group is associated with adverse outcomes similar to those experienced by older adults, including hospitalization, nursing home admission, and death. Developing ADL impairment in middle age also impacts work force participation and health expenditures, not just in middle age but also older age. Middle-aged adults have a high capacity for recovery from functional impairment and many risk factors for functional impairment have their roots in middle age. Taken together, these findings suggest that middle age may be an ideal period to intervene to prevent or delay functional impairment. Multi-pronged efforts are needed to address the rising prevalence of functional impairment in middle age. These include developing improved prognostic tools to identify middle-aged people at highest risk for functional impairment and adapting existing interventions or developing novel interventions to prevent or delay functional impairment in this age group. Exercise interventions may be particularly effective for middle-aged people whose main risk factor for functional impairment is declining strength and physical performance; for more socioeconomically vulnerable populations, multi-component interventions that address health, social, and environmental risk factors may be needed. In addition, we need to shift our health system towards recognizing functional impairment in middle age as a problem that is as prevalent and central to health and wellbeing as many chronic medical conditions.

## Data Availability

Not applicable.

## Abbreviations

ADL: Activities of daily living
IADL: Instrumental activities of daily living
LIFE: Lifestyle Interventions and Independence for Elders
CAPABLE: Community Aging in Place – Advancing Better Living for Elders
